# Global Spread of Mutant PfCRT and Its Pleiotropic Impact on Plasmodium falciparum Multidrug Resistance and Fitness

**DOI:** 10.1128/mBio.02731-18

**Published:** 2019-04-30

**Authors:** Satish K. Dhingra, Stanislaw J. Gabryszewski, Jennifer L. Small-Saunders, Tomas Yeo, Philipp P. Henrich, Sachel Mok, David A. Fidock

**Affiliations:** a Department of Microbiology and Immunology, Columbia University Irving Medical Center, New York, New York, USA; b Division of Infectious Diseases, Department of Medicine, Columbia University Medical Center, New York, New York, USA; NIAID, NIH

**Keywords:** *Plasmodium falciparum*, drug resistance evolution, fitness, malaria, *pfcrt*

## Abstract

The global spread of Plasmodium falciparum chloroquine resistance transporter (PfCRT) variant haplotypes earlier caused the widespread loss of chloroquine (CQ) efficacy. In Asia, novel PfCRT mutations that emerged on the Dd2 allelic background have recently been implicated in high-level resistance to piperaquine, and N326S and I356T have been associated with genetic backgrounds in which resistance emerged to artemisinin derivatives. By analyzing large-scale genome sequencing data, we report that the predominant Asian CQ-resistant Dd2 haplotype is undetectable in Africa. Instead, the GB4 and previously unexplored Cam783 haplotypes predominate, along with wild-type, drug-sensitive PfCRT that has reemerged as the major haplotype. To interrogate how these alleles impact drug susceptibility, we generated *pfcrt*-modified isogenic parasite lines spanning the mutational interval between GB4 and Dd2, which includes Cam783 and involves amino acid substitutions at residues 326 and 356. Relative to Dd2, the GB4 and Cam783 alleles were observed to mediate lower degrees of resistance to CQ and the first-line drug amodiaquine, while resulting in higher growth rates. These findings suggest that differences in growth rates, a surrogate of parasite fitness, influence selection in the context of African infections that are frequently characterized by high transmission rates, mixed infections, increased immunity, and less recourse to treatment. We also observe that the Asian Dd2 allele affords partial protection against piperaquine yet does not directly impact artemisinin efficacy. Our results can help inform the regional recommendations of antimalarials, whose activity is influenced by and, in certain cases, enhanced against select PfCRT variant haplotypes.

## Abstract (precis)

Our study defines the allelic distribution of *pfcrt*, an important mediator of multidrug resistance in Plasmodium falciparum, in Africa and Asia. We leveraged whole-genome sequence analysis and gene editing to demonstrate how current drug combinations can select different allelic variants of this gene and shape region-specific parasite population structures. We document the ability of PfCRT mutations to modulate parasite susceptibility to current antimalarials in dissimilar, *pfcrt* allele-specific ways. This study underscores the importance of actively monitoring *pfcrt* genotypes to identify emerging patterns of multidrug resistance and help guide region-specific treatment options.

## INTRODUCTION

The pathogenesis of human malaria is initiated by *Plasmodium* asexual blood-stage parasites, with Plasmodium falciparum causing the most severe and lethal forms of disease. Recent reductions in the worldwide incidence of malaria can be largely attributed to the global deployment of artemisinin (ART)-based combination therapies (ACTs) to treat asexual blood-stage infections, combined with vector control strategies (1). ACTs consist of a fast-acting, rapidly eliminated ART compound, paired with a long-lived partner drug necessary to clear residual parasite biomass. P. falciparum resistance to certain ACT drugs has emerged in Southeast (SE) Asia, raising concerns that it might soon extend to Africa where malaria exerts its greatest impact (2, 3). This pattern of emerging resistance would mimic the prior spread from Asia to Africa of P. falciparum resistance to the former first-line drugs chloroquine (CQ) and pyrimethamine (4, 5). The global threat posed by multidrug resistance underscores the pressing need to characterize its genetic determinants and leverage that knowledge into improved methods of surveillance, containment, and treatment.

Artemether plus lumefantrine (ATM+LMF), artesunate plus amodiaquine (AS+ADQ), and dihydroartemisinin plus piperaquine (DHA+PPQ) are the three ACTs used most widely worldwide (2). The structural hallmark of these ART derivatives is an endoperoxide bridge that, upon cleavage by parasite-processed host heme-bound Fe^2+^, is potently parasiticidal. Once activated, ARTs can reduce the parasite biomass by up to 10^4^-fold per ∼48-h cycle of intraerythrocytic development (6). Clinically, emerging resistance to ARTs is defined as extended parasite clearance times in AS- or ACT-treated patients, first reported in western Cambodia and now increasingly observed in neighboring countries (7–12). Slower clearance is associated with single nucleotide polymorphisms (SNPs) in the propeller domain of the P. falciparum *k13* (*kelch13*) gene (13, 14). These mutations, including the most widespread C580Y mutation, as well as R539T, were shown in gene-edited parasites to confer ART resistance *in vitro* (15, 16). Resistance *in vitro* is defined as ≥1% survival of early ring-stage parasites (0 to 3 h postinvasion [hpi]) exposed for 6 h to the ART metabolite DHA, tested at the pharmacologically relevant concentration of 700 nM (ring-stage survival assay from 0 to 3 h [RSA_0-3h_]) (17). Molecular epidemiological and clinical data show that mutant K13 parasites are now widespread throughout SE Asia, but to date, they remain infrequent in Africa where ACTs currently retain excellent efficacy (9, 10, 18, 19).

The evolution of K13-mediated ART resistance in SE Asia has been attributed, in part, to the presence of a favorable genetic background that permits sustained parasite transmission in the field. Through a multicenter genome-wide association study, founder populations were identified that included mutations in parasite genes encoding the P. falciparum chloroquine resistance transporter (PfCRT), the apicoplast ribosomal protein S10, ferredoxin, and the P. falciparum multidrug resistance 2 transporter (20). Prior transfection-based studies have demonstrated that some PfCRT mutations can modestly modulate parasite ART susceptibility *in vitro*, as determined in 72-h dose-response assays that measure half-maximal growth inhibition concentrations (IC_50_ values) (21, 22). PfCRT mutations also impact the intracellular disposition of heme, which serves as the ART activator (23–25). PfCRT mutations are also known to modulate the potency of several ACT partner drugs, including LMF, mefloquine (MFQ), ADQ, and PPQ (2, 21, 26–31). Their selective pressures can therefore influence the regional prevalence of PfCRT haplotypes.

The role of mutant PfCRT in dictating P. falciparum resistance to the former first-line antimalarial CQ, along with the pleotropic impact of this transporter on multiple other drugs, has driven a detailed assessment of PfCRT evolution and its impact on drug resistance and fitness (22, 32). First discovered through the analysis of a P. falciparum genetic cross between the CQ-resistant Dd2 line (from SE Asia) and the CQ-sensitive HB3 line (from Honduras), mutant isoforms have been discovered to harbor four to nine SNPs, depending on their geographic origin (26, 33). Earlier studies of African isolates observed the FCB isoform, harboring seven mutations compared to the CQ-sensitive canonical 3D7 wild-type allele, along with a 6-amino-acid variant termed GB4 (Table 1) (26, 34, 35). These studies relied on samples that predate the use of ACTs in the region. Epidemiological reports have often examined only positions 72 to 76 in exon 2 of the *pfcrt* gene, without assigning complete haplotypes (36). Thus, the current distribution of *pfcrt* alleles in Africa and Asia, post-ACT treatment selection, has remained largely unknown.

**TABLE 1:** PfCRT and K13 haplotypes of recombinant isogenic parasite lines^*a*^

Parasite line	Amino acid in PfCRT at the following position:								Amino acid in K13 at position 539
	74	75	76	220	271	326	356	371	
Dd2^3D7^ (wild type)	M	N	K	A	Q	N	I	R	R
Dd2^Dd2^	**I**	**E**	**T**	**S**	**E**	**S**	**T**	**I**	R
Dd2^Cam783^ (Dd2^Dd2 S326N^)	**I**	**E**	**T**	**S**	**E**	N	**T**	**I**	R
Dd2^FCB^ (Dd2^Dd2 T356I^)	**I**	**E**	**T**	**S**	**E**	**S**	I	**I**	R
Dd2^GB4^ (Dd2^Dd2 S326N T356I^)	**I**	**E**	**T**	**S**	**E**	N	I	**I**	R
Dd2_R539T_^3D7^	M	N	K	A	Q	N	I	R	**T**
Dd2_R539T_^Dd2^	**I**	**E**	**T**	**S**	**E**	**S**	**T**	**I**	**T**
Dd2_R539T_^Cam783^ (Dd2_R539T_^Dd2 S326N^)	**I**	**E**	**T**	**S**	**E**	N	**T**	**I**	**T**
Dd2_R539T_^FCB^ (Dd2_R539T_^Dd2 T356I^)	**I**	**E**	**T**	**S**	**E**	**S**	I	**I**	**T**
Dd2_R539T_^GB4^ (Dd2_R539T_^Dd2 S326N T356I^)	**I**	**E**	**T**	**S**	**E**	N	I	**I**	**T**

a The mutational status of PfCRT (PlasmoDB PF3D7_0709000) and K13 (PF3D7_1343700) is summarized for all recombinant parasite lines generated in this study. Mutations are shown in boldface type. The PfCRT haplotype encoded by Dd2 (wild-type K13) and Dd2_R539T_ (K13 R539T mutant) parasites is indicated in superscript, with 3D7 designating the wild-type PfCRT haplotype.

Genetic and mathematical modeling studies that deconstructed the evolution of *pfcrt* alleles provided evidence that mutations within this gene likely occurred in bursts, with the preferred paths representing a balance between gaining stepwise resistance to CQ and minimizing the negative impact of reduced fitness, as determined from differences in parasite asexual blood-stage growth rates (22). An important role for fitness was highlighted by the finding in Malawi that complete removal of CQ pressure led to the virtual disappearance of mutant PfCRT as a result of it causing a decreased proliferation rate and the dominance of faster-growing wild-type parasites in these settings of frequent mixed infections (37, 38).

In this study, we leveraged recent advances in whole-genome sequence analysis of P. falciparum isolates across multiple sites in Asia and Africa (9) to explore the geographic distribution of *pfcrt* alleles and determine their impacts on asexual blood-stage parasite drug resistance and fitness, including the reported association of the PfCRT mutations N326S and I356T with emerging ART resistance (20). Our findings present novel insights into PfCRT haplotype distributions across Asia and Africa and how they might be impacted by regional differences in drug selection and parasite fitness.

## RESULTS

### Distribution of *pfcrt* alleles in Africa and Asia.

**FIG 1:**
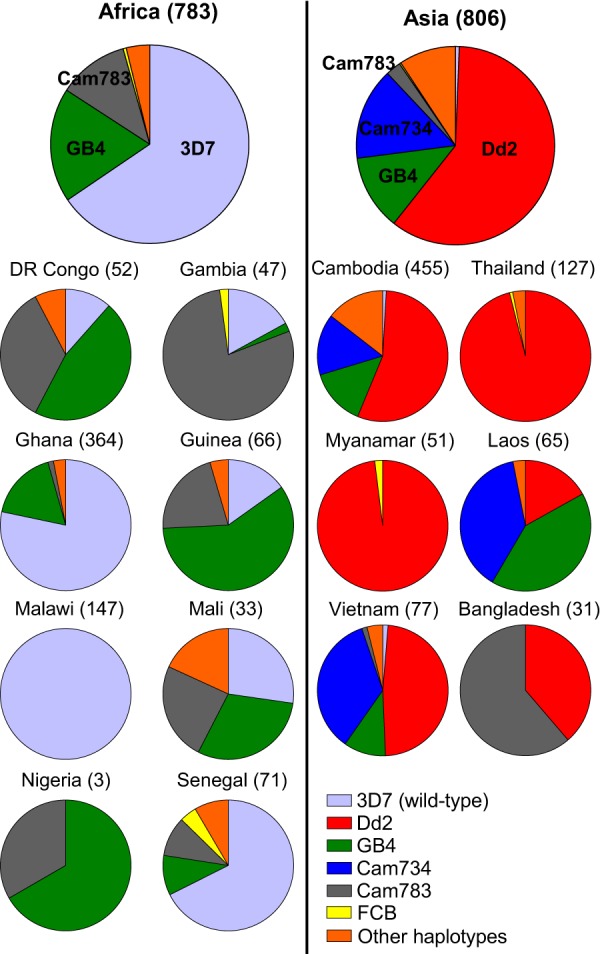
Predominant *pfcrt* alleles are distinct in Africa and Asia. Pie charts illustrate continent- and country-wide proportions of the indicated PfCRT haplotypes, as estimated based on whole-genome sequences present in the MalariaGEN Pf3k database (9). Analysis revealed the following haplotype proportions: in Africa, 3D7 (65.5%), GB4 (18.6%), Cam783 (11.5%), FCB (0.5%), other (3.8%); in SE Asia, 3D7 (0.6%), Dd2 (60%), GB4 (12.3%), Cam734 (15%), Cam783 (2.5%), FCB (0.2%), other (9.3%). Country-wide distribution of the *pfcrt* alleles is detailed in Tables S1 and S2 in the supplemental material. The mutational composition of the wild-type (3D7) and variant PfCRT haplotypes is shown in Table 1. Cam734 PfCRT consists of the following mutations: M74I, N75D, K76T, A144F, L148I, I194T, A220S, Q271E, and T333S.

Although sub-Saharan Africa accounts for the vast majority of the global burden of malaria, SE Asia is critical in driving much of the emergence of multidrug resistance. Using the MalariaGEN Pf3k database of recently acquired P. falciparum whole-genome sequences that encompass these regions (9), we assessed the geographic distribution of *pfcrt* haplotypes by region and sampled country (Fig. 1 and Table 1; see also Tables S1, S2, and S3 in the supplemental material). In Africa, wild-type (3D7) PfCRT comprised 65.5% of the total haplotypes (513/783 genomes), consistent with reduced CQ use and the increased fitness of wild-type PfCRT in the absence of drug selection. Countries showed widely different percentages of wild-type PfCRT ranging from 100% in Malawi (all 147 genomes) to 11.5% in the Democratic Republic of Congo (6 of 52 genomes). These findings could be explained in part by differences in the local availability and use of CQ.

10.1128/mBio.02731-18.5 TABLE S1 Frequency distribution of PfCRT haplotypes from the Pf3K data set across Africa. Download Table S1, PDF file, 0.02 MB. Copyright © 2019 Dhingra et al. 2019 Dhingra et al. This content is distributed under the terms of the Creative Commons Attribution 4.0 International license.

10.1128/mBio.02731-18.6 TABLE S2 Frequency distribution of PfCRT haplotypes from the Pf3K data set across Asia. Download Table S2, PDF file, 0.02 MB. Copyright © 2019 Dhingra et al. 2019 Dhingra et al. This content is distributed under the terms of the Creative Commons Attribution 4.0 International license.

10.1128/mBio.02731-18.7 TABLE S3 PfCRT haplotype distribution across Asia and Africa, as derived from the Pf3K data set. Download Table S3, PDF file, 0.1 MB. Copyright © 2019 Dhingra et al. 2019 Dhingra et al. This content is distributed under the terms of the Creative Commons Attribution 4.0 International license.

We found no evidence of the 8-amino-acid variant Dd2 haplotype in Africa (Fig. 1, Table 1, and Table S1). Instead, GB4 was the most common African mutant PfCRT haplotype (18.6% total prevalence, observed in 146 of 783 genomes), followed by the Cam783 haplotype (akin to Dd2 minus the N326S mutation; 11.5% of total, 90/783 genomes). Of note, not a single report has until now examined the Cam783 haplotype in parasites. The remaining portion of mutant haplotypes (4% of total) was comprised of various polymorphic haplotypes, with the FCB haplotype (akin to Dd2 minus the I356T mutation) being present at 0.5% prevalence (4 of 783 genomes). Among the remaining genomes, the genomes with reduced numbers of mutations (GB4-N75E and GB4-A220S) were the most common, suggesting selection against the more mutated isoforms (Table S3).

In Asia, the wild-type PfCRT haplotype had virtually disappeared, comprising only 0.6% of the total haplotypes (5/806 genomes) despite the removal of CQ pressure at least 20 years ago for the treatment of P. falciparum malaria in favor of more recent ACTs (39). Dd2 was the most common mutant haplotype (60.0% of total, 484/806 genomes), followed by the 9-amino-acid variant Cam734 (15.0% of total, 121/806 genomes), GB4 (12.3% of total, 99/806 genomes), Cam783 (2.5%; 20/806 genomes), and other mutant haplotypes (9.3% of total, 75/806 genomes; Tables S2 and S3).

### Generation of isogenic parasites with variant *pfcrt* and *k13* alleles.

In view of the highly significant association between the PfCRT mutations N326S and I356T and the emergence of ART resistance in SE Asia (with a *P* value of 7 × 10^−10^) (20), we tested whether these mutations could individually or collectively influence antimalarial susceptibility and/or parasite fitness in a way that would explain their local dominance across SE Asia. We note that these mutations are found together only in the Dd2 haplotype (Table 1 and Fig. 1). Using an established zinc-finger nuclease (ZFN)-based approach (Fig. S1), we generated isogenic parasites differing only by their *pfcrt* allele. The *pfcrt* sequences of these parasite lines were verified using primers listed in Table S4. Our PfCRT haplotype panel included full-length 3D7 (wild type), Dd2, GB4 (Dd2 minus the N326S and I356T mutations), and the two intermediates spanning the mutational interval between GB4 and Dd2, namely, the Cam783 allele (Dd2 minus the N326S mutation) and FCB (Dd2 minus the I356T mutation; see Table 1). Inclusion of the Cam783 haplotype allowed us to characterize the second most common mutant PfCRT haplotype in Africa (Fig. 1). We generated this panel of PfCRT haplotypes in two separate parasite backgrounds, namely, Dd2 and Dd2_R539T_, which encode the wild-type K13 (default) and R539T mutant isoforms (shown as a subscript), respectively (Table 1). The latter strain was previously reported as Dd2^R539T^, whose K13 mutation conferred one of the highest levels of ART resistance in Dd2 parasites, exceeding that of the more prevalent K13 C580Y mutation. We opted to use Dd2^R539T^ in our studies as it therefore maximized our ability to measure small changes in the ring-stage survival assay (RSA) levels with different PfCRT haplotypes compared with wild-type K13 parasites (16). We included a K13 variant line in light of the rapid spread of mutant K13 across SE Asia (10) and the need to assess whether mutant K13 might alter the pleotropic impact of PfCRT on antimalarial drug susceptibility.

10.1128/mBio.02731-18.2 FIG S1 Allelic modification of *pfcrt* and validation of genetic editing. (A) Zinc-finger nuclease (ZFN)-based genetic engineering strategy. As detailed in Text S1, parasites were first enriched with a donor plasmid (p*crt*-h*dhfr*) that encodes *pfcrt* exons 2 to 13 (e2-13) and includes mutations of interest (indicated in red). A comprehensive list of PfCRT haplotypes that were introduced into Dd2 or Dd2_R539T_ parasites is found in Table 1. The p*crt*-h*dhfr* plasmid also comprises the following elements: Plasmodiumberghei *crt* (*pbcrt*) 3′ UTR, human *dhfr* (h*dhfr*) selection cassette, as well as *pfcrt-hdhfr*-flanking homology regions (∼0.4 kb upstream of the intron 1-exon 2 junction and ∼1 kb native 3′ UTR downstream of *hdhfr*). Parasites were subsequently transfected with pZFN*^crt^*-*bsd*. This plasmid facilitates *calmodulin* (*cam*) promoter-driven expression of a pair of *pfcrt*-targeting ZFNs (ZFN_L_ and ZFN_R_) and includes the *blasticidin S deaminase* (*bsd*) selection cassette. Following ZFN-mediated introduction of a double-stranded break in *pfcrt* (indicated with a lightning bolt), parasites that employ the p*crt*-h*dhfr* plasmid as a donor template incorporate mutations of interest through homologous recombination-based mechanisms. Genetic editing was evaluated by diagnostic PCR (see panel B), and successfully edited parasites were cloned by limiting dilution. (B) Blood PCR-based verification of *pfcrt* allelic exchange. Shown are representative results for Dd2 parasites into which the full-length GB4 *pfcrt* allele was introduced (Dd2^GB4^) as well as controls, namely, genetically unedited parental parasites (Dd2), unedited donor plasmid-enriched parasites (Dd2 + p*crt*-h*dhfr*), and the sole donor plasmid (p*crt*-h*dhfr*). All appropriately edited lines showed the expected DNA band migration patterns: 0.4 kb (p7+p8), 1.2 kb (p9+p8), 2.5 kb (p10+p11), and 2.7 kb (p9+p12). Primer (p) locations are illustrated in panel A. Download FIG S1, EPS file, 1.9 MB. Copyright © 2019 Dhingra et al. 2019 Dhingra et al. This content is distributed under the terms of the Creative Commons Attribution 4.0 International license.

10.1128/mBio.02731-18.1 TEXT S1 Supplemental Materials and Methods. Download Text S1, PDF file, 0.2 MB. Copyright © 2019 Dhingra et al. 2019 Dhingra et al. This content is distributed under the terms of the Creative Commons Attribution 4.0 International license.

10.1128/mBio.02731-18.8 TABLE S4 Primers used in this study. Download Table S4, PDF file, 0.02 MB. Copyright © 2019 Dhingra et al. 2019 Dhingra et al. This content is distributed under the terms of the Creative Commons Attribution 4.0 International license.

### CQ responses of *pfcrt*-modified lines and their chemosensitization by verapamil.

**FIG 2:**
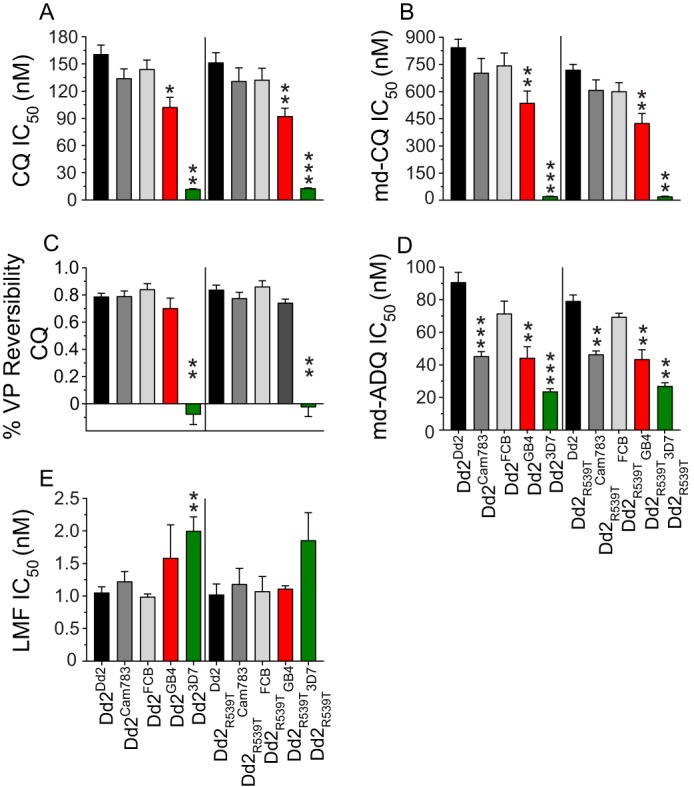
Responses of *pfcrt*-modified lines to various antimalarial drugs. Parasite susceptibilities to chloroquine (CQ), monodesethyl-CQ (md-CQ), md-amodiaquine (md-ADQ), and lumefantrine (LMF) were assessed by flow cytometry following exposure to the indicated drug for 72 h. (A, B, D, and E) Bar graphs show mean plus SEM IC_50_ values. (C) Percent CQ reversibility was determined as follows: 1 − CQ response modification index (RMI), with RMI being the IC_50_ value for CQ in the presence of 800 nM VP divided by the IC_50_ value for CQ alone. The bar graph shows mean plus SEM reversibility values. For all panels, results reflect 3 to 10 independent assays conducted in duplicate. Statistical significance was determined against Dd2^Dd2^ or Dd2_R539T_^Dd2^ by two-tailed Mann-Whitney *U* tests using Graphpad Prism 7 software. *, *P* < 0.05; **, *P* < 0.01; ***, *P* < 0.001.

To assess the impact of these *pfcrt* alleles on parasite CQ responses, we subjected asexual blood-stage parasites to flow cytometry-based drug susceptibility assays and determined 50% and 90% growth-inhibitory antimalarial drug concentrations (IC_50_ and IC_90_, respectively). For CQ and its main *in vivo* metabolite, monodesethyl-CQ (md-CQ) (Fig. 2A and B; Table S5), mutant PfCRT isoforms Dd2, Cam783, FCB, and GB4 each conferred statistically significant increases in IC_50_ values compared with the isogenic comparator line expressing the wild-type 3D7 isoform (*P < *0.002 in all instances by two-tailed Mann-Whitney *U* tests). Among the mutant isoforms, the highest and lowest levels of resistance to CQ and md-CQ were conferred by the Dd2 and GB4 PfCRT isoforms, respectively (Fig. 2A and B; Table S5). The reduced level of resistance associated with GB4 PfCRT (∼1.6-fold reduction versus Dd2 PfCRT for CQ and md-CQ) was significant in both the Dd2 and Dd2_R539T_ parasite genetic backgrounds (*P < *0.01 by two-tailed Mann-Whitney *U* tests; Fig. 2A and B; Table S5). These data suggest that GB4 might represent a mutational precursor of Dd2 PfCRT and that the latter proved more successful in the epidemiological context of SE Asia.

10.1128/mBio.02731-18.9 TABLE S5 Antimalarial IC_50_ and IC_90_ values of *pfcrt*-modified parasite lines. Download Table S5, PDF file, 0.3 MB. Copyright © 2019 Dhingra et al. 2019 Dhingra et al. This content is distributed under the terms of the Creative Commons Attribution 4.0 International license.

Chemosensitization of CQ-resistant parasites by the calcium channel blocker verapamil (VP) is a defining feature of parasite CQ resistance (40). VP reversibility can be quantified as the CQ response modification index (RMI), corresponding to the following equation: 1 − (IC_50_ value for CQ in the presence of 0.8 μM VP/IC_50_ value for CQ alone) (41). Comparison of the RMI values for our isogenic *pfcrt*-modified lines (Fig. 2C; Table S5) showed that the Dd2, Cam783, FCB, and GB4 isoforms yielded similar levels of VP reversibility, contrasting with isogenic parasites expressing the 3D7 isoform that showed no VP chemosensitization (21). Our observed CQ, md-CQ, and VP responses were equivalent between the *k13* wild-type Dd2 and *k13* mutant Dd2_R539T_ lines.

### Susceptibility of *pfcrt*-modified lines to clinically employed antimalarials.

We also examined our isogenic, *pfcrt*-modified lines for their susceptibility to a panel of clinically important drugs, namely, the ACT artemisinin compound AS, various ACT partner drugs (ADQ, PPQ, LMF, and pyronaridine [PND]), and the quinoline-type drug quinine (QN) (Fig. 2D and E; Fig. S2 and Table S5). All mutant *pfcrt* alleles conferred a degree of parasite resistance to the active ADQ metabolite md-ADQ compared with 3D7 *pfcrt*, with Dd2 *pfcrt* being the most resistant (Fig. 2D; Table S5). Interestingly, an important contributory role was identified for the N326S mutation. Mutant PfCRT haplotypes bearing S326N reversions (i.e., Cam783 and GB4; see Table 1) conferred a statistically significant reduction in md-ADQ resistance relative to the Dd2 isoform (*P < *0.001 for Dd2 versus Cam783 and *P < *0.01 for Dd2 versus GB4 by two-tailed Mann-Whitney *U* tests; Fig. 2D and Table S5). The notion that PfCRT residue 326 is an important contributor to ADQ resistance agrees with recent evidence that the N326D mutation, found among South American and western Pacific parasite isolates, contributes directly to md-ADQ resistance (22). We also observed a statistically significant ∼2-fold increase in the LMF IC_50_ value in the 3D7 allele compared to the Dd2 isoform in the K13 wild-type Dd2 background (Fig. 2E; Table S5). No statistically significant differences were observed between individual mutant PfCRT haplotypes for the compounds AS, PPQ, LMF, QN, and PND (Fig. 2E; Fig. S2 and Table S5).

10.1128/mBio.02731-18.3 FIG S2 Drug susceptibility of *pfcrt*-modified lines to antimalarial drugs. Parasite susceptibilities to artesunate (AS), piperaquine (PPQ), quinine (QN), and pyronaridine (PND) were assessed after 72-h exposures to the indicated drug. Parasitemia was measured using flow cytometry. Data are presented as mean ± SEM IC_50_ values for 3 to 10 independent assays performed in duplicate. Statistical significance was determined against Dd2^Dd2^ or Dd2_R539T_^Dd2^ by two-tailed Mann-Whitney *U* tests using Graphpad Prism 7 software. Download FIG S2, EPS file, 0.8 MB. Copyright © 2019 Dhingra et al. 2019 Dhingra et al. This content is distributed under the terms of the Creative Commons Attribution 4.0 International license.

### Survival of *k13*-mutant *pfcrt*-modified lines exposed to DHA.

Although our drug susceptibility assays did not reveal physiologic significant shifts in AS IC_50_ values among parasite lines, the utility of IC_50_-based studies for the detection of clinical ART resistance is restricted. A more clinically validated metric is parasite survival following drug pulses, which more closely mimics the *in vivo* pharmacology (17). For ART compounds, reduced susceptibility of early ring-stage parasites (0 to 3 hpi) to a 6-h pulse of 700 nM DHA in the *in vitro* RSAs correlates with ART resistance in the clinical setting, defined as lower rates of parasite clearance (17, 42). This phenotype has been associated with mutations in the parasite *k13* gene, which is sufficient to mediate enhanced survival of DHA-pulsed parasites in RSAs (13, 16).

To examine whether PfCRT mutations could modulate the parasite’s ability to survive DHA pulses, we employed the *k13*-mutant Dd2_R539T_ background (Table 1), which bears a K13 R539T mutation that confers *in vitro* RSA survival to DHA. We also included the ART-resistant mutant *k13* parasite line Cam3.II_R539T_, which carries the PfCRT Dd2 haplotype as a positive control (16). We also considered the possibility that PfCRT mutations may modulate ART resistance at subphysiologic levels of DHA in a stage-specific manner. Accordingly, we evaluated the survival of early ring (0 to 3 h) and mid-trophozoite (28 to 31 h) blood-stage parasites following exposure to a wide range of DHA concentrations (twofold dilutions spanning the range of 700 nM to 1.4 nM).

**FIG 3:**
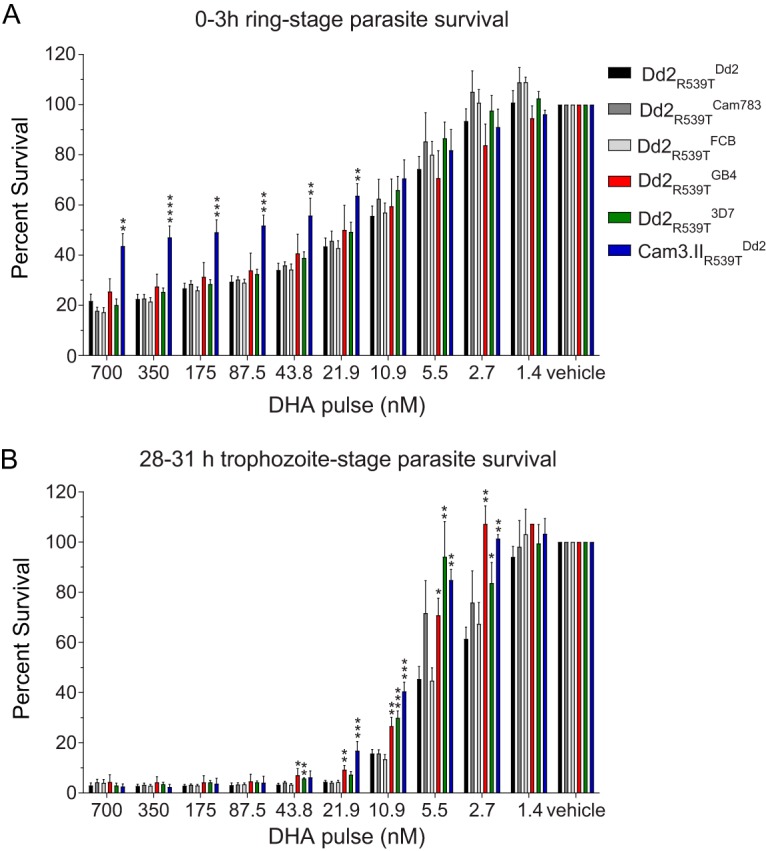
Dihydroartemisinin (DHA) survival of *k13*-mutant, *pfcrt*-modified lines. Tightly synchronized parasites at the early ring (0 to 3 hpi) (A) or mid-trophozoite (28 to 31 hpi) (B) developmental stage were exposed to a 3-h pulse of DHA at the indicated concentrations. Parasite viability was determined by flow cytometry at 72 hpi. Bar graphs correspond to the mean plus SEM percent survival, equivalent to the parasitemia of DHA-treated parasites divided by the parasitemia of vehicle control (DMSO)-treated parasites. Data are from three or four independent experiments, conducted in duplicate. Statistical significance was determined by two-tailed Student’s *t* tests (Graphpad Prism 7). *, *P* < 0.05; **, *P* < 0.01; ***, *P* < 0.001.

Results showed no impact of varying the PfCRT haplotype on parasite survival for any concentration examined (Fig. 3A). In contrast, in trophozoite-stage DHA survival assays (Fig. 3B), statistically significant differences were observed at the subphysiologic concentrations of 43.8 nM to 2.7 nM DHA. Parasites with 3D7 and GB4 PfCRT exhibited significantly increased protection from DHA toxicity compared to parasites with either Dd2 or FCB allele (*P < *0.05 and *P < *0.001 as determined by two-tailed Student’s *t* test). Compared to ring-stage parasites, trophozoites were considerably more susceptible to DHA exposure (e.g., compare parasite survival for 700 nM DHA in Fig. 3A and for 10.9 nM DHA in Fig. 3B, which show comparable survival rates). The highly DHA-resistant Cam3.II_R539T_ showed >40% survival in the ring stage, consistent with an earlier report (16), yet conferred no appreciable survival at the trophozoite stage.

### Survival of PPQ-exposed *k13*-mutant *pfcrt*-modified lines.

Similar to ART resistance, IC_50_-based drug susceptibility studies appear insufficient to reveal parasite resistance to the first-line ACT partner drug PPQ. This is apparent from *ex vivo* analyses of recrudescent parasites derived from DHA+PPQ treatment failures, which show a wide range of PPQ IC_50_ values that overlap with IC_50_ values of nonresistant isolates (43). Recent studies of PPQ-resistant parasites have highlighted the effectiveness of *in vitro* PPQ survival assays (PSAs) in uncovering resistance phenotypes that correlate with *in vivo* parasite recrudescence (43). In these assays, synchronized early ring-stage parasites are subjected to 200 nM PPQ for 48 h. Drug is then removed by washing, and parasite survival is assessed at the 72-h endpoint. The basis for a much longer duration of drug exposure compared to DHA-based assays is the relatively prolonged half-life of PPQ in blood following treatment (44).

**FIG 4:**
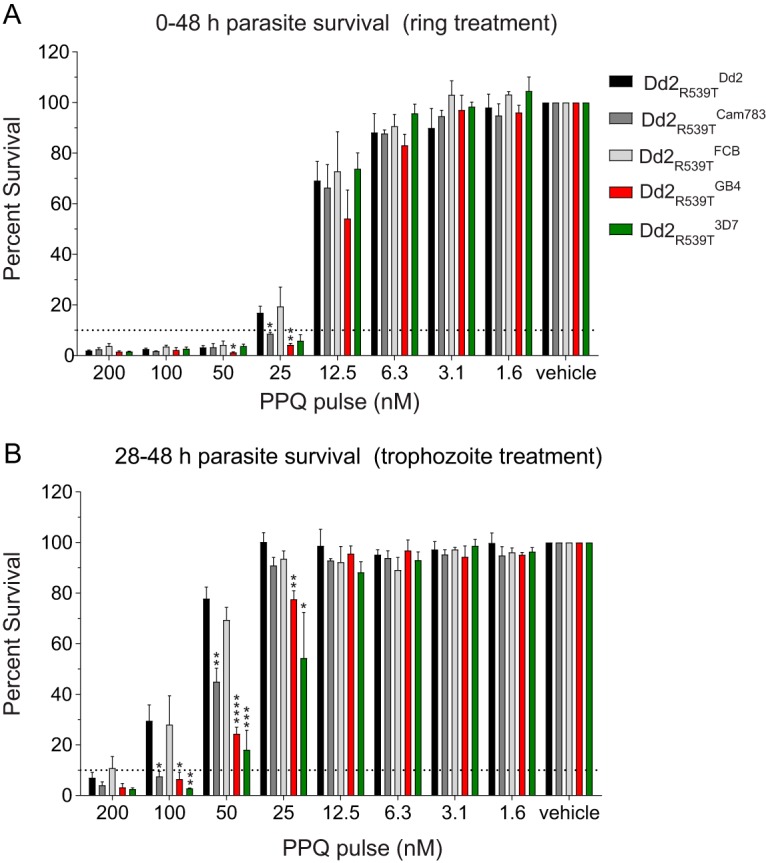
Piperaquine (PPQ) survival of *k13*-mutant, *pfcrt*-modified lines. Tightly synchronized parasites in the early ring (0 to 3 hpi) (A) or mid-trophozoite (28 to 31 hpi) (B) developmental stage were incubated at the indicated doses of PPQ until 48 hpi, after which drug was washed out. Parasite viability was determined by flow cytometry at 72 hpi. Bar graphs correspond to the mean plus SEM percent survival, equivalent to the parasitemia of PPQ-treated parasites divided by the parasitemia of vehicle control (0.5% lactic acid)-treated parasites. Data are from two to four independent experiments, conducted in duplicate. Statistical significance was determined by two-tailed Student’s *t* tests (Graphpad Prism 7). *, *P* < 0.05; **, *P* < 0.01; ***, *P* < 0.001.

Similar to our DHA-based survival assays, we subjected *pfcrt*-modified Dd2_R539T_ parasites to serial dilutions of PPQ starting from a maximum concentration of 200 nM. To probe for stage-specific effects, we exposed parasites to PPQ at either the early ring stage (0 to 3 hpi) or mid-trophozoite stage (28 to 31 hpi). Results for PPQ survival assays initiated during the early ring stage showed increased susceptibility of parasites expressing the *pfcrt* GB4 allele at 50 nM and 25 nM PPQ compared to isogenic parasites expressing the Dd2 allele (Fig. 4A; *P < *0.05 and *P < *0.01, respectively, as determined by two-tailed Student’s *t* test). At 25 nM PPQ concentration, our PSA data showed no significant differences between the Dd2 and FCB alleles and an increased PPQ susceptibility with the Cam783 allele (*P < *0.05). These results implicate residue 326 as a contributor to increased survival to PPQ at lower concentrations.

Intriguingly, for PSAs initiated during the mid-trophozoite stage, when expression of PfCRT is maximal, we observed increased PPQ susceptibilities with the Cam783, GB4, and 3D7 alleles compared to the Dd2 allele at 100 nM, 50 nM, and 25 nM PPQ (Fig. 4B). The increased PPQ susceptibility at both the ring and trophozoite stages of the Cam783 allele, compared with the isogenic Dd2 *pfcrt*-expressing allele, suggests a role for the N326S mutation (present in Dd2) in reducing susceptibility to PPQ (Fig. 4B; *P < *0.05 and *P < *0.01 at 100 nM and 50 nM, respectively, as determined by two-tailed Student’s *t* test). We did not observe any differences in the survival of parasites with the FCB and Dd2 alleles, arguing against a role for residue 356 in modulating parasite susceptibility to PPQ (Fig. 4B).

### *In vitro* growth of *pfcrt*-modified lines.

*Plasmodium* fitness is a multifaceted property that reflects the reproductive success of parasites over multiple rounds of infection. Previous analyses of isogenic parasites expressing variant *pfcrt* alleles have shed light on their ability to influence growth rates and have uncovered important inferences regarding their global spread (22, 45). Here, we used an established flow cytometry-based coculture assay that compares growth of green fluorescent protein (GFP)-negative (GFP^–^) *pfcrt*-modified test lines with that of a GFP-positive (GFP^+^) reporter line (22) (see Materials and Methods). The GFP^–^ proportion of coculture was regularly determined for 10 parasite generations and then used to derive the per-generation selection coefficient (*s*) associated with a given *pfcrt* allele. This parameter was used as a reflection of asexual blood-stage fitness, with *s *=* *0, *s *>* *0, and *s *<* *0 indicating fitness equal to, greater than, and less than that of Dd2^3D7^ parasites (i.e., parasites with wild-type alleles at both the *pfcrt* and *k13* loci).

**FIG 5:**
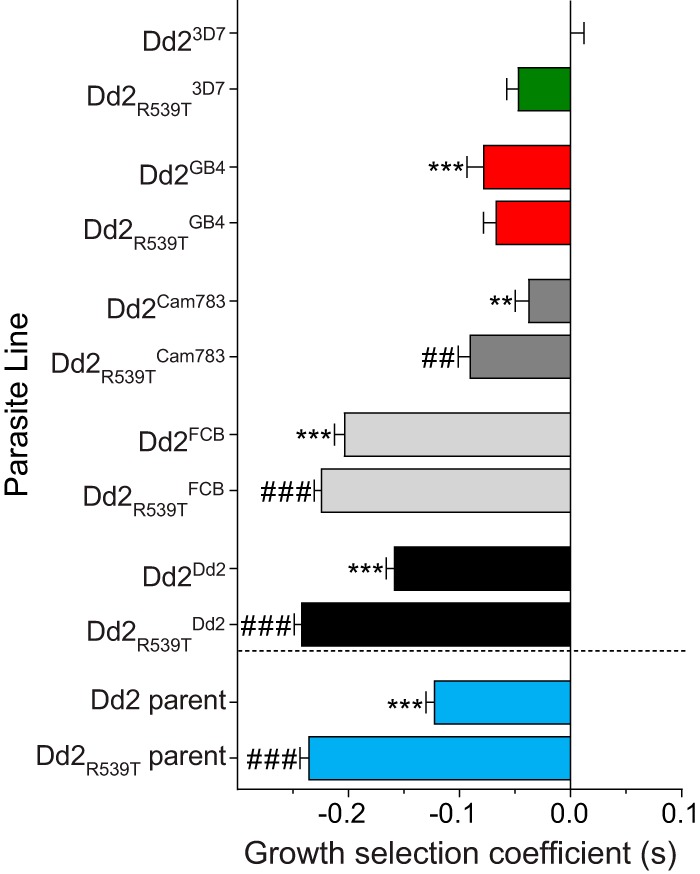
*In vitro* growth characteristics of *pfcrt*-modified and reference parasite lines. Cocultures consisting of a 1:1 ratio of the GFP^–^ test strain and a GFP^+^ reporter strain (R^GFP^) were initiated at day 0 and were monitored by flow cytometry for 10 generations. The per-generation selection coefficient (*s*) for each test strain was determined as described in Text S1 and reflects parasite growth compared to Dd2^3D7^ parasites, which carry the wild-type (3D7) *pfcrt* allele (i.e., *s *=* *0 for Dd2^3D7^). The bars depict mean plus SEM *s* values (detailed in Table S6), as determined in three independent assays, conducted in duplicate. Statistical significance between different PfCRT haplotypes was determined against Dd2^3D7^ (**, P < 0.01; ***, *P* *< *0.001) or Dd2_R539T_^3D7^ (##, *P < *0.01; ###, *P < *0.001) by two-way ANOVA with Sidak’s *post hoc* test using GraphPad Prism 7 software.

Our results revealed several key differences in the fitness costs associated with mutant *pfcrt* alleles (Fig. 5; Fig. S3 and Table S6). Among PfCRT-matched lines expressing the 3D7, Cam783, or Dd2 haplotype, those harboring mutant R539T K13 showed a more pronounced, statistically significant growth defect compared to parasites with wild-type K13 (compare panels A and B in Fig. S3). In both the Dd2 and Dd2_R539T_ genetic backgrounds, the Dd2 and *pfcrt* FCB alleles both conferred a notable fitness cost (range of mean *s* values, −0.16 to −0.24) compared with wild-type 3D7. Interestingly, the *pfcrt* GB4 and Cam783 alleles were associated with a less severe fitness cost (range of mean *s* values, −0.09 to −0.04) compared with the Dd2 and FCB alleles. Given that GB4 and Cam783 are the two predominant mutant PfCRT isoforms found in Africa (Fig. 1), this finding implicates fitness as a driver of haplotype selection in this region (46).

10.1128/mBio.02731-18.4 FIG S3 Fitness assay of *pfcrt*-modified lines. *In vitro* growth, used as a proxy of fitness of *pfcrt*-modified lines in wild-type Dd2 K13 (A) or a K13 R539T (B) genetic background. Parasite lines were cocultured with a GFP^+^ reporter Dd2 line in equal proportions for a period of 20 days (10 parasite generations). Parasitemia was measured using flow cytometry. Data are presented as means ± SEM from three independent experiments performed in duplicate. Download FIG S3, EPS file, 0.9 MB. Copyright © 2019 Dhingra et al. 2019 Dhingra et al. This content is distributed under the terms of the Creative Commons Attribution 4.0 International license.

10.1128/mBio.02731-18.10 TABLE S6 *In vitro* growth selection coefficients of *pfcrt*-modified and reference parasite lines. Download Table S6, PDF file, 0.1 MB. Copyright © 2019 Dhingra et al. 2019 Dhingra et al. This content is distributed under the terms of the Creative Commons Attribution 4.0 International license.

## DISCUSSION

By leveraging a large collection of whole-genome sequence data from African and Asian P. falciparum isolates, our study presents new insights into the global distribution of *pfcrt* alleles. In Africa, we observe that two-thirds of the sampled isolates now carry wild-type, CQ-sensitive *pfcrt*, with the CQ-resistant alleles being predominantly GB4 and Cam783. Notably, the Cam783 allele has previously not been reported in Africa, nor has it been dissected for its drug susceptibility profile. The presence of the Cam783 allele in Africa (ranging between 10% in Senegal to 79% in The Gambia; Fig. 1; see also Table S1 in the supplemental material) also indicates a possible migratory event from Asia to Africa that had remained undetected due to the low numbers of isolates sampled in prior studies (34). Alternatively, Cam783 might have evolved from the Dd2 allele via loss of the single N326S mutation, resulting in an allele that maintained a high level of CQ resistance and relatively improved parasite fitness (Fig. 2 and 5; Fig. S3 and Table S1).

The FCB allele, which was earlier suggested to have originally migrated from SE Asia to Africa (26, 34, 35), was observed at a prevalence of only 0.5% in our cohort of 783 genomes. Our analysis of African and Asian parasite genomes suggests that GB4 might have been an original migratory haplotype or might have evolved from FCB in Africa (via loss of the N326S mutation) and spread as a result of GB4’s improved fitness.

Using ZFN-based gene editing, we investigated the pleiotropic roles of PfCRT amino acid positions 326 and 356, which separate the majority of haplotypes circulating in Africa or Asia. Results show that the GB4 haplotype, which is wild type at these residues, is less resistant to CQ, md-CQ, and the ADQ metabolite md-ADQ compared to Dd2. The African Cam783 haplotype that is wild type at residue 326 also showed significant decrease in md-ADQ resistance compared to Dd2. Both the GB4 and Cam783 haplotypes nonetheless conferred higher md-ADQ IC_50_ values compared to the drug-sensitive wild-type 3D7 haplotype.

Most *pfcrt* alleles increased parasite susceptibility to LMF, as previously observed with the Dd2 haplotype and in agreement with clinical reports showing that LMF tends to select against *pfcrt* variant alleles in favor of the wild-type allele (27, 36, 47). Our drug susceptibility profiling data support the use of both ATM+LMF and AS+ADQ in regions of endemicity, as they appear to exert opposing selective pressure on *pfcrt* mutant and wild-type alleles, thus creating bottlenecks that could suppress multidrug resistance.

Earlier studies with isogenic, *pfcrt*-modified parasites support the notion that Dd2 PfCRT confers a substantial fitness cost to parasites, compared with the Cambodian Cam734 and South American/western Pacific 7G8 isoforms (45). Our current data suggest that parasites expressing Dd2 PfCRT persist in SE Asia, but not in Africa. Their persistence in Asia might be caused in part by the continued local use of CQ to treat Plasmodiumvivax infections, producing sufficient drug pressure to also select for mutant *pfcrt* in individuals exposed to both plasmodial species. Another explanation could be the reduced use of LMF in most countries in SE Asia, thus reducing selection pressure against Dd2 *pfcrt*. SE Asia also experiences a much lower level of overall transmission compared to Africa, translating in the former case to fewer mixed infections and greater population reliance on the use of drugs to resolve P. falciparum infections. We speculate that the epidemiological situation in Africa, where asymptomatic adults can frequently harbor multiple strains and use antimalarials relatively infrequently, provides for within-host selection that favors rapidly proliferating strains and selects against strains where resistance places them at a competitive growth disadvantage.

One impetus for our study was the report that the PfCRT N326S and I356T mutations present in Dd2 were associated with K13 mutations that have emerged in areas of emerging ART resistance (20). Therefore, we also constructed these mutant *pfcrt* alleles in a *k13* mutant genetic background (K13 R539T) that affords elevated ART resistance, as defined using the *in vitro* RSA with very young rings. Our results showed no protective effect of mutant PfCRT in ring-stage survival, including at the 700 nM DHA concentration that has been validated as a surrogate for *in vitro* resistance and clinical efficacy (17). When tested with trophozoites, we observed that the *pfcrt* wild-type and GB4 alleles provided a small but significant survival advantage at lower concentrations of DHA compared to the Dd2 allele (Fig. 3B).

These findings suggest that the appearance in Asia of K13 mutations on the Dd2 background, beginning in Cambodia, might relate to other aspects of PfCRT function. For instance, ART toxicity to *Plasmodium* parasites is thought to possibly involve the induction of oxidative stress, as suggested by prior real-time studies of the parasite glutathione (GSH)-dependent redox state (48). Given the prospect that GSH is potentially one of the substrates of PfCRT (49, 50), it is plausible that the Dd2 form of PfCRT may help P. falciparum avert oxidative damage, perhaps through PfCRT-mediated transport of GSH into the parasite digestive vacuole (DV), where GSH has been proposed to degrade toxic heme (51, 52). Alternatively, these associations might simply reflect the initial emergence of mutant K13 on founder populations that harbored Dd2 PfCRT, whose N326S and I356T mutations are absent in the other common Asian haplotypes Cam734 and GB4 (Fig. 1 and Table 1).

Hemoglobin catabolism and heme detoxification, which occur primarily in the parasite’s DV and which furnish the parasite with its primary source of amino acids, are central to the modes of action and/or resistance to a wide array of ACT drugs (6, 23, 53, 54). PfCRT’s presence as a transporter on the DV membrane, where it helps regulate multiple physiological parameters, including hemoglobin-derived peptide and heme levels, DV pH, and ion homeostasis, in addition to its capacities to mediate drug efflux, position it to directly or indirectly alter interactions of multiple ACT drugs with heme moieties or hemozoin. Of these, the ability of certain PfCRT variant haplotypes to mediate parasite resistance to the CQ-like bisquinoline PPQ is of particular concern, as recent reports document a rapid emergence and spread of PPQ resistance. DHA+PPQ treatment failure rates exceeding 50% have now been reported in western Cambodia (55–58). Recent gene editing data, including Cambodian isolates, now provide compelling evidence that several novel *pfcrt* alleles that emerged on the Dd2 background have rapidly increased in prevalence in the past few years and can contribute to high-level PPQ resistance (30). These alleles were observed in parasites harboring multiple copies of the hemoglobinases plasmepsin II and III, with this gene amplification previously identified as a marker of PPQ-resistant parasites (59–62). Evidence suggests that plasmepsin II and III amplification might correct for fitness costs observed in *pfcrt* variants, exemplified by their abnormal DV morphologies and presumably a result of impaired peptide processing and efflux out of the DV (30). These novel *pfcrt* alleles are all derived from the Dd2 haplotype and include the additional mutations H97Y, F145I, and G353V. The use of Dd2 as the founder haplotype is consistent with our findings that this isoform mediates the highest level of parasite survival in PSAs conducted with trophozoites (Fig. 4B). Our study helps explain this finding by showing a role for the N326S mutation in particular in augmenting PSA survival beginning with trophozoites as well as ring stages. These data emphasize the value of delineating molecular markers that can help identify and contain the spread of PPQ resistance in SE Asia.

ADQ is a second ACT partner drug of particular interest with regard to mutant *pfcrt* alleles given the widespread use of AS+ADQ for the treatment and prevention of malaria in Africa. ADQ also comprises the formulation ADQ plus sulfadoxine-pyrimethamine, which is administered prophylactically to young children in some areas of sub-Saharan Africa during months of high transmission of malaria. Recent clinical studies have documented a lower incidence of parasite recrudescence following treatment with AS+ADQ compared to ATM+LMF, the two most widely deployed regimens that are known to select for mutant and wild-type *pfcrt*-expressing parasites, respectively (63). Based on the wide prevalence of the wild-type *pfcrt* allele in Africa and the paucity of *pfcrt* alleles that conferred a high degree of ADQ resistance in our study, the continued use of ADQ-based regimens in Africa seems justified. A meta-analysis of worldwide AS+ADQ efficacy showed a sevenfold-greater risk of parasite recrudescence after AS+ADQ treatment in Asia compared to Africa (64). Given these data, it is possible that only certain *pfcrt* alleles such as Dd2 are able to confer the minimum degree of ADQ resistance necessary to sustain resistant parasite populations, whereas other mutant *pfcrt* alleles (e.g., GB4 and Cam783) are more rapidly cleared and, in the context of appropriate dosing, pose less of a threat.

As malaria control efforts intensify in the coming years, their success will depend, in part, on the precise definition and monitoring of markers of parasite multidrug resistance. Our study supports the notion of PfCRT being a pleiotropic mediator of parasite multidrug resistance (21, 27, 33, 65, 66). The results suggest that opposing selective forces can be found in single ACT formulations; for example, in our survival assays, DHA and PPQ provided some evidence of selection for K76 and K76T, respectively (Fig. 3B and 4B). Likewise, these opposing selective forces may also be exerted by the long-lived partner drugs comprising distinct ACT formulations, such as PPQ or ADQ, in particular regions. Consistent with this, we document the ability of PfCRT mutations to modulate parasite LMF, ADQ, and PPQ susceptibility in dissimilar, *pfcrt* allele-specific ways. Our findings support the practice of antimalarial regimen cycling and underscore the importance of active monitoring of *pfcrt* genotypes to identify emerging patterns of multidrug resistance and help guide region-specific treatment options.

## MATERIALS AND METHODS

### Parasite genetic modification and cultivation.

Details regarding our ZFN-based *pfcrt* modification strategy, verification of appropriate genetic editing, and isolation of genetically modified parasite clones are found in Fig. S1 and Text S1 in the supplemental material. Unless stated otherwise, P. falciparum*-*infected human erythrocytes were cultured at 4% hematocrit in RPMI 1640 culture medium supplemented with 0.5% Albumax II (Invitrogen) (67). Cultures were incubated at 37°C in 5% O_2_/5% CO_2_/90% N_2_.

### Analysis of MalariaGEN Pf3K field isolate genome data.

*pfcrt* sequence analyses were performed on genome data generated by the Plasmodium falciparum Community Genome Project (https://www.malariagen.net/projects/pf3k; pilot data release 3). This data set comprised 2,512 samples from 14 countries, acquired mostly in 2011-2012 (9). Assembly files were downloaded from the publicly accessible database ftp://ngs.sanger.ac.uk/production/pf3k/release_3/3.1/ , along with an additional 87 genomes obtained from Cambodian isolates in 2012 and 2013 deposited at the European Bioinformatics Institute (9, 20). SNPs were manually extracted using the Pf3D7 reference genome version 11.0 as previously described (68). Briefly, we identified all SNPs and rare variants present in the *pfcrt* genes of the field isolates based on the criteria of an alternate allele frequency of >0.4. To assign the correct PfCRT haplotypes, we filtered out samples where the alternate allele count at 12 major codon positions (74, 75, 76, 144, 148, 194, 220, 271, 326, 333, 356, and 371) was below 5. This yielded 783 African and 806 Asian isolates that were analyzed.

### Drug susceptibility assays.

The concentrations of antimalarial drugs that were 50% and 90% growth inhibitory (i.e., IC_50_ and IC_90_) to asexual blood-stage parasites were determined using a flow cytometry-based assay that detects parasite nuclei and mitochondria using SYBR Green I and MitoTracker Deep Red, respectively (69). Parasites were subjected to treatment with drug (twofold serial dilutions of drug), and parasite growth was determined with an Accuri C6 flow cytometer after 72 h. Assays were performed with CQ with or without 0.8 μM VP, md-CQ, md-ADQ, AS, PPQ, LMF, QN, and PND. To determine VP-mediated reversibility of CQ resistance, the CQ+VP IC_50_ value was divided by the CQ IC_50_ value, yielding the CQ response modification index (RMI) (41). VP reversibility was defined as 1 − CQ RMI value. Statistical significance was determined via nonparametric, two-tailed Mann-Whitney *U* tests using GraphPad Prism 7 software.

### DHA and PPQ survival assays.

Stage-specific survival assays were conducted following published protocols, with minor modifications (16, 17, 43, 70). Briefly, 15 ml of parasite culture was synchronized with 5% sorbitol and cultivated through the mature schizont stage (≥0.5% schizont parasitemia). Cultures were heparinized for 30 min at 37°C in RPMI 1640 medium containing 15 U/ml sodium heparin (Millipore) with occasional mild vortexing. Parasites were subsequently subjected to a gradient of 75% Percoll (Sigma-Aldrich) and centrifuged at 3,400 rpm for 15 min. The schizont fraction was washed with RPMI 1640 medium and incubated with human red blood cells (RBCs) for 3 h to initiate a new asexual blood-stage cycle, followed by synchronization with 5% sorbitol to eliminate non-ring parasite forms. Serial dilutions of DHA or PPQ were prealiquoted into 96-well plates using a BioTek precision pipetting system. Parasite cultures at early ring (0 to 3 hpi) or mid-trophozoite (18 to 21 hpi) stages were added to plates containing drug dilutions, at 0.5% parasitemia, 2% hematocrit, and 200-μl final volume per well. Duplicate wells were included for each parasite line and drug concentration. Vehicle controls for DHA and PPQ survival assays were DMSO and 0.5% lactic acid, respectively. For the RSA_0-3h_, parasites were exposed to 700 nM DHA for 3 h. For the PSA_0-3h_, parasites were exposed to 200 nM PPQ for 48 h. Following drug incubation, parasites were washed three times with RPMI 1640 medium and cultured in fresh medium until the assay endpoint of 72 h. Parasitemia was assessed by flow cytometry, which correlated with microscopic assessment of parasitemia. Parasite survival was calculated as the parasitemia in the presence of drug divided by the parasitemia in the vehicle control well, expressed as a percentage.

### *In vitro* growth assays.

Growth of asexual blood-stage parasites was assessed *in vitro* using a previously described method that employs flow cytometric detection of GFP and the far-red fluorescent dye SYTO 61 (22). The latter was used to further separate infected from uninfected red blood cells. Briefly, 1:1 cocultures were established with a GFP-positive (GFP^+^) fluorescent reporter line (R^GFP^) and a single GFP-negative (GFP^–^) *pfcrt*-modified test line. R^GFP^ in these assays corresponds to the previously generated CQ-resistant Dd2^BiP-eGFP^ line (71). This line uses a BiP promoter to drive enhanced GFP (eGFP) expression and has a genetic background comparable to that of our *pfcrt*-modified test lines. Using flow cytometry, we calculated the proportion of GFP^–^ parasites (corresponding to the *pfcrt*-modified test line) every 3 days for 20 days (i.e., 10 asexual blood-stage parasite generations). Cultures were regularly monitored and diluted such that parasitemias never exceeded 8%. Per-generation selection coefficients (*s*) associated with *pfcrt* alleles were calculated as described in Text S1. Statistical significance was assessed via two-way ANOVA with Sidak’s *post hoc* test using GraphPad Prism 7 software.
