# Supplementary material for: Global Spread of Mutant PfCRT and Its Pleiotropic Impact on Plasmodium falciparum Multidrug Resistance and Fitness
Source: mBio. 2019 Apr 30;10(2):e02731-18. doi: 10.1128/mBio.02731-18 (PMC6495381; doi:10.1128/mBio.02731-18)
Supplement: TABLE S1 [file mBio.02731-18-st001.pdf]

Supplementary Table S1. Frequency distribution of PfCRT haplotypes from the Pf3K dataset across Africa.

**Isoform**	**# of variants**	**No. of isolates**	**Total %**	**DR of the Congo**	**%**	**Ghana**	**%**	**Guinea**	**%**	**Malawi**	**%**	**Mali**	**%**	**Nigeria**	**%**	**Senegal**	**%**	**The Gambia**	**%**
3D7 (wild-type)	0	513	65.5%	6	11.5%	285	78.3%	10	15.2%	147	100.0%	9	27.3%	-	-	48	67.6%	8	17.0%
Dd2	8	-	-	-	-	-	-	-	-	-	-	-	-	-	-	-	-	-	-
GB4	6	146	18.6%	24	46.2%	63	17.3%	39	59.1%	-	-	10	30.3%	2	66.7%	7	9.9%	1	2.1%
Cam734	9	-	-	-	-	-	-	-	-	-	-	-	-	-	-	-	-	-	-
Cam783	7	90	11.5%	18	34.6%	5	1.4%	14	21.2%	-	-	8	24.2%	1	33.3%	7	9.9%	37	78.7%
FCB	7	4	0.5%	-	-	-	-	-	-	-	-	-	-	-	-	3	4.2%	1	2.1%
Other haplotypes		30	3.8%	4	7.7%	11	3.0%	3	4.5%	-	-	6	18.2%	-	-	6	8.5%	-	-
Total		783	100.0%	52		364		66		147		33		3		71		47	
Percentage of total				6.6%		46.5%		8.4%		18.8%		4.2%		0.4%		9.1%		6.0%	

Samples were collected from the Pf3K data version 3 (totaling 783 genomes that passed our analysis). A dash indicates that the allele was not observed in any genomes from that country.
