# Supplementary material for: Global Spread of Mutant PfCRT and Its Pleiotropic Impact on Plasmodium falciparum Multidrug Resistance and Fitness
Source: mBio. 2019 Apr 30;10(2):e02731-18. doi: 10.1128/mBio.02731-18 (PMC6495381; doi:10.1128/mBio.02731-18)
Supplement: TABLE S2 [file mBio.02731-18-st002.pdf]

Supplementary Table S2. Frequency distribution of PfCRT haplotypes from the Pf3K dataset across Asia.

Isoform	# of variants	No. of isolates	Total %	Cambodia	%	Laos	%	Myanmar	%	Thailand	%	Vietnam	%	Bangladesh	%
3D7 (wild-type)	0	5	0.6%	4	0.9%	-	-	-	-	-	-	1	1.3%	-	-
Dd2	8	484	60.0%	252	55.4%	11	16.9%	50	98.0%	122	96.1%	37	48.1%	12	38.7%
GB4	6	99	12.3%	64	14.1%	27	41.5%	-	-	-	-	8	10.4%	-	-
Cam734	9	121	15.0%	69	15.2%	25	38.5%	-	-	-	-	27	35.1%	-	-
Cam783	7	20	2.5%	-	-	-	-	-	-	-	-	1	1.3%	19	61.3%
FCB	7	2	0.2%	-	-	-	-	1	2.0%	1	0.8%	-	-	-	-
Other haplotypes		75	9.3%	66	14.5%	2	3.1%	-	-	4	3.1%	3	3.9%	-	-
Total		806	100.0%	455		65		51		127		77		31	
Percentage of total				56.5%		8.1%		6.3%		15.8%		9.6%		3.8%	

Samples were collected from the Pf3K data version 3 and Cambodian genomes collected in 2012 and 2013 that were more recently deposited by the Pf3K consortium totalling 806 genomes that passed our analysis. A dash indicates that the allele was not observed in any genomes from that country.
