# Supplementary material for: Global Spread of Mutant PfCRT and Its Pleiotropic Impact on Plasmodium falciparum Multidrug Resistance and Fitness
Source: mBio. 2019 Apr 30;10(2):e02731-18. doi: 10.1128/mBio.02731-18 (PMC6495381; doi:10.1128/mBio.02731-18)
Supplement: TABLE S3 [file mBio.02731-18-st003.pdf]

Supplementary Table S3. PfCRT haplotype distribution across Asia and Africa, as derived from the Pf3K dataset.

			No. of isolates		Asia (per country sampled)						Africa (per country sampled)							
**Haplotype**	**PfCRT Haplotype**	**No. variants**	**ASIA**	**AFRICA**	**Cambodia**	**Laos**	**Myanmar**	**Thailand**	**Vietnam**	**Bangladesh**	**DR Congo**	**Ghana**	**Guinea**	**Malawi**	**Mali**	**Nigeria**	**Senegal**	**The Gambia**
3D7 (wild-type)	3D7 (wild-type)	-	5	513	4	-	-	-	1	-	6	285	10	147	9	-	48	8
Dd2	M74I;N75E;K76T;A220S;Q271E;N326S;I356T;R371I	8	484	-	252	11	50	122	37	12	-	-	-	-	-	-	-	-
GB4	M74I;N75E;K76T;A220S;Q271E;R371I	6	99	146	64	27	-	-	8	-	24	63	39	-	10	2	7	1
Cam734	M74I;N75D;K76T;A144F;L148I;I194T;A220S;Q271E;T333S	9	121	-	69	25	-	-	27	-	-	-	-	-	-	-	-	-
Cam783	M74I;N75E;K76T;A220S;Q271E;I356T;R371I	7	20	90	-	-	-	-	1	19	18	5	14	-	8	1	7	37
FCB	M74I;N75E;K76T;A220S;Q271E;N326S;R371I	7	2	4	-	-	1	1	-	-	-	-	-	-	-	-	3	1
Cam738	M74I;N75D;K76T;L148I;I194T;A220S;Q271E;T333S	8	-	-	-	-	-	-	-	-	-	-	-	-	-	-	-	-
GB4 + I194T (Novel)	M74I;N75E;K76T;I194T;A220S;Q271E;R371I	7	9	-	7	2	-	-	-	-	-	-	-	-	-	-	-	-
GB4 - N75E (Novel)	M74I;K76T;A220S;Q271E;R371I	5	3	13	1	-	-	-	2	-	2	8	3	-	-	-	-	-
GB4 - M74I - N75E (Novel)	K76T;A220S;Q271E;R371I	4	-	1	-	-	-	-	-	-	-	-	-	-	1	-	-	-
GB4 - A220S (Novel)	M74I;N75E;K76T;Q271E;R371I	5	2	7	2	-	-	-	-	-	1	3	-	-	-	-	3	-
Dd2 + T93S (Novel)	M74I;N75E;K76T;T93S;A220S;Q271E;N326S;I356T;R371I	9	1	-	1	-	-	-	-	-	-	-	-	-	-	-	-	-
Dd2 + H97L (Novel)	M74I;N75E;K76T;H97L;A220S;Q271E;N326S;I356T;R371I	9	7	-	6	-	-	1	-	-	-	-	-	-	-	-	-	-
Dd2 + H97Y (Novel)	M74I;N75E;K76T;H97Y;A220S;Q271E;N326S;I356T;R371I	9	15	-	15	-	-	-	-	-	-	-	-	-	-	-	-	-
Dd2 + F145I (Novel)	M74I;N75E;K76T;F145I;A220S;Q271E;N326S;I356T;R371I	9	-	-	-	-	-	-	-	-	-	-	-	-	-	-	-	-
Dd2 + L196P (Novel)	M74I;N75E;K76T;L196P;A220S;Q271E;N326S;I356T;R371I	9	3	-	-	-	-	3	-	-	-	-	-	-	-	-	-	-
Dd2 + I218F (Novel)	M74I;N75E;K76T;I218F;A220S;Q271E;N326S;I356T;R371I	9	10	-	10	-	-	-	-	-	-	-	-	-	-	-	-	-
Dd2 + T256I (Novel)	M74I;N75E;K76T;A220S;T256I;Q271E;N326S;I356T;R371I	9	5	-	5	-	-	-	-	-	-	-	-	-	-	-	-	-
Dd2 + N295I (Novel)	M74I;N75E;K76T;A220S;Q271E;N295I;N326S;I356T;R371I	9	-	-	-	-	-	-	-	-	-	-	-	-	-	-	-	-
Dd2 + M343L (Novel)	M74I;N75E;K76T;A220S;Q271E;N326S;M343L;I356T;R371I	9	3	-	3	-	-	-	-	-	-	-	-	-	-	-	-	-
Dd2 + G353V (Novel)	M74I;N75E;K76T;A220S;Q271E;N326S;G353V;I356T;R371I	9	8	-	8	-	-	-	-	-	-	-	-	-	-	-	-	-
Dd2 + A366T (Novel)	M74I;N75E;K76T;A220S;Q271E;N326S;I356T;A366T;R371I	9	2	-	2	-	-	-	-	-	-	-	-	-	-	-	-	-
Dd2 + G367C (Novel)	M74I;N75E;K76T;A220S;Q271E;N326S;I356T;G367C;R371I	9	2	-	2	-	-	-	-	-	-	-	-	-	-	-	-	-
Dd2 + V370E (Novel)	M74I;N75E;K76T;A220S;Q271E;N326S;I356T;V370C;R371I	9	-	-	-	-	-	-	-	-	-	-	-	-	-	-	-	-
Dd2 - N75E (Novel)	M74I;K76T;A220S;Q271E;N326S;I356T;R371I	7	2	-	1	-	-	-	1	-	-	-	-	-	-	-	-	-
Dd2 - A220S (Novel)	M74I;N75E;K76T;Q271E;N326S;I356T;R371I	7	3	-	3	-	-	-	-	-	-	-	-	-	-	-	-	-
Cam738 + N75E (Novel)	M74I;N75E;K76T;L148I;I194T;A220S;Q271E;T333S	8	-	-	-	-	-	-	-	-	-	-	-	-	-	-	-	-
Cam783-N75E (Novel)	M74I;K76T;A220S;Q271E;I356T;R371I	6	-	6	-	-	-	-	-	-	1	-	-	-	2	-	3	-
Cam783-M74I-N75E (Novel)	K76T;A220S;Q271E;I356T;R371I	5	-	3	-	-	-	-	-	-	-	-	-	-	3	-	-	-
Cam783+V141L (Novel)	M74I;N75E;K76T;V141L;A220S;Q271E;I356T;R371I	8	-	-	-	-	-	-	-	-	-	-	-	-	-	-	-	-
		Total	806	783	455	65	51	127	77	31	52	364	66	147	33	3	71	47

Samples were collected from the Pf3K data version 3 totaling 806 genomes from Southeast Asia and 783 from Africa that passed our filtering criteria including Cambodian genomes collected in 2012 and 2013 that were more recently deposited by the Pf3K consortium. A dash indicates that the allele was not observed in any genomes from that country. Filtering criteria retained genomes only if the allelic balance for each nucleotide in the pfcrt coding sequence was > 0.4 and in the case of mutant alleles if the alternative coverage was ≥ 5.
