# Supplementary material for: Global Spread of Mutant PfCRT and Its Pleiotropic Impact on Plasmodium falciparum Multidrug Resistance and Fitness
Source: mBio. 2019 Apr 30;10(2):e02731-18. doi: 10.1128/mBio.02731-18 (PMC6495381; doi:10.1128/mBio.02731-18)
Supplement: TEXT S1 [file mBio.02731-18-s0001.pdf]

Supplementary Materials and Methods

Genetic Modification of the Parasite *pfcrt* Locus

To genetically modify the *pfcrt* locus of Dd2 or $Dd2_{R539T}$ parasites, a set of p*crt*-h*dhfr* donor plasmids encoding variant *pfcrt* alleles was prepared. This set included the previously generated plasmids pcrt^{3D7}-h*dhfr* and pcrt^{Dd2}-h*dhfr* (*pfcrt* allele listed in superscript; see **Table 1**). Site-directed mutagenesis with *Pfu*Turbo Polymerase (Agilent) and primers pairs p1+p2 and p3+p4 was used to introduced PfCRT back-to-wildtype S326N and/or T356I mutations into the donor template pcrt^{Dd2}-h*dhfr*, generating the additional donor plasmids p$crt^{Dd2\ S326N}$-h*dhfr*, p$crt^{Dd2\ T356I}$-h*dhfr*, and pcrt^{GB4}-h*dhfr*.

Our ZFN-based genetic engineering strategy is detailed in **Fig. S1A**. Briefly, Dd2 or $Dd2_{R539T}$ parasites (≥4% parasitemia, mostly ring-stage) were transfected with 40 µg donor plasmid (p*crt*-h*dhfr*) and pressured with 2.5 nM WR99210 (Jacobus Pharmaceuticals). Parasites enriched with the p*crt*-h*dhfr* plasmid were similarly transfected with pZFN^{crt}-*bsd* and pressured with 2.5 nM WR99210 and 2 µg/ml Blasticidin HCl (Invitrogen) for a period of six days, followed by continuous pressure with 2.5 nM WR99210. As detailed elsewhere (1), blood PCR reactions (KAPA Biosystems; shown in **Fig. S1B**) were used to assess for successful allelic replacement in bulk cultures and in cloned recombinant parasites. Parasites were cloned by limiting dilution following established protocols (2). The full-length *pfcrt* gene was PCR-amplified with primer pair p9+p12 and sequenced with primers p5 and p6 to verify sequence integrity.

Determination of *In Vitro* Growth Selection Coefficients

Parasite growth in co-cultures consisting of a GFP^{-} test line and a GFP^{+} reporter line was monitored regularly for 10 generations (detailed in **Materials and Methods**). The frequencies of

the GFP$^-$ test line and the GFP$^+$ reporter line at time t are referred to as p_t and q_t, respectively. As previously detailed, the fitness (ω) associated with a particular test line was derived from the natural log-transformed ratio p_t/q_t and normalized to isogenic parasites encoding the wild-type (3D7) *pfcrt* allele in the Dd2 genetic background (Dd2^{3D7}), yielding the relative fitness (ω') metric (3). The *in vitro* growth selection coefficient (s) for each test strain was derived from the relationship $s = \omega' - 1$ (4). Statistical significance was assessed via two-way ANOVA with Sidak's post-hoc test using GraphPad Prism 7 software.

Supplementary References

1. Gabryszewski SJ, Modchang C, Musset L, Chookajorn T, Fidock DA. 2016. Combinatorial genetic modeling of *pfcrt*-mediated drug resistance evolution in *Plasmodium falciparum*. Mol Biol Evol 33:1554-70. https://doi.org/10.1093/molbev/msw037.
2. Adjalley SH, Lee MC, Fidock DA. 2010. A method for rapid genetic integration into *Plasmodium falciparum* utilizing mycobacteriophage Bxb1 integrase. Methods Mol Biol 634:87-100. https://doi.org/10.1007/978-1-60761-652-8_6.
3. Gabryszewski SJ, Dhingra SK, Combrinck JM, Lewis IA, Callaghan PS, Hassett MR, Siriwardana A, Henrich PP, Lee AH, Gnadig NF, Musset L, Llinas M, Egan TJ, Roepe PD, Fidock DA. 2016. Evolution of fitness cost-neutral mutant PfCRT conferring *P. falciparum* 4-aminoquinoline drug resistance Is accompanied by altered parasite metabolism and digestive vacuole physiology. PLoS Pathog 12:e1005976. https://doi.org/10.1371/journal.ppat.1005976.
4. Baker S, Duy PT, Nga TV, Dung TT, Phat VV, Chau TT, Turner AK, Farrar J, Boni MF. 2013. Fitness benefits in fluoroquinolone-resistant *Salmonella* Typhi in the absence of antimicrobial pressure. Elife 2:e01229. https://doi.org/10.7554/eLife.01229.
