# Supplementary material for: Global Spread of Mutant PfCRT and Its Pleiotropic Impact on Plasmodium falciparum Multidrug Resistance and Fitness
Source: mBio. 2019 Apr 30;10(2):e02731-18. doi: 10.1128/mBio.02731-18 (PMC6495381; doi:10.1128/mBio.02731-18)
Supplement: TABLE S4 [file mBio.02731-18-st004.pdf]

Supplementary Table S4. Primers used in this study.

Primer	Oligonucleotide sequence (5' → 3')	Description	Lab name
p1	ccttcgcattgttttccttctttaAcatttgtgataatttaataaccagc	SDM PfCRT S326N F	p1765
p2	GCTGGTTATTAAATTATCACAAATGTTAAAGAAGGAAAACAATGCGAAGG	SDM PfCRT S326N F	p1766
p3	GTTAGTTGTATACAAGGTCCAGCAATAGCAATTGCTTATTAC	SDM PfCRT T356I F	p5633
p4	GTAATAAGCAATTGCTATTGCTGGACCTTGTATACAACTAAC	SDM PfCRT T356I R	p5634
p5	TCAAACATGACAAGGGAAATAGT	*pfcrt* exon 5 F	p2427
p6	CCAAGAATAAACATGCGAAACC	*pfcrt* exon 7 R	p3806
p7	CTTGAATTCGACCTTAACAGATGGCTCAC	*pfcrt* exon 2 F	p3264
p8	CTTATCGATAAGCAGAAGAACATATTAATAGGAATACTTAATTG	*pfcrt* exon 3 R	p3265
p9	CTTGGGCCCAAGTTGTACTGCTTCTAAGC	*pfcrt* gDNA 5′ UTR F	p3404
p10	CTCGAGATGGTTGGTTCGCTAAACTGC	h*DHFR* F	p3315
p11	TTGACCCTTATATATTCCACCCA	*pfcrt* gDNA 3′ UTR R	p3403
p12	GAGGCGCCTATTTCAAAAATCTTAGCATAAGGATT	*pbcrt* 3′ UTR R	p1644

SDM, site-directed mutagenesis; F, forward; R, reverse; gDNA, genomic DNA; *pbcrt, P. berghei chloroquine resistance transporter*; UTR, untranslated region.
