# Supplementary material for: Global Spread of Mutant PfCRT and Its Pleiotropic Impact on Plasmodium falciparum Multidrug Resistance and Fitness
Source: mBio. 2019 Apr 30;10(2):e02731-18. doi: 10.1128/mBio.02731-18 (PMC6495381; doi:10.1128/mBio.02731-18)
Supplement: TABLE S5 [file mBio.02731-18-st005.pdf]

Supplementary Table S5 (page 1 of 2). Antimalarial IC_{50} and IC_{90} values of *pfcrt*-modified parasite lines.

	$Dd2^{Dd2}$	$Dd2^{Cam783}$	$Dd2^{FCB}$	$Dd2^{GB4}$	$Dd2^{3D7}$	$Dd2_{R539T}^{Dd2}$	$Dd2_{R539T}^{Cam783}$	$Dd2_{R539T}^{FCB}$	$Dd2_{R539T}^{GB4}$	$Dd2_{R539T}^{3D7}$
**CQ IC_{50}**	161 ± 10.1	134 ± 10.5	144 ± 10.1	102 ± 10.8	12.1 ± 0.4	152 ± 10.8	131 ± 14.6	132 ± 12.9	92.5 ± 8.8	12.9 ± 0.5
n	7	7	5	4	6	7	6	5	4	7
P	–	0.05	0.27	0.01	0.0012	–	0.23	0.27	0.0061	0.0006
**CQ IC_{90}**	261 ± 12.0	231 ± 9.9	268 ± 27.6	204 ± 18.1	18.4 ± 1.9	277 ± 24.4	255 ± 17.8	263 ± 26.9	199 ± 13.3	24.9 ± 0.7
n	7	7	5	4	6	7	6	5	4	7
P	–	0.10	0.64	0.01	0.0012	–	0.18	0.2	0.0061	0.0006
**CQ+VP IC_{50}**	32.5 ± 4.1	26.6 ± 4.6	22.5 ± 5.8	30.7 ± 8.9	12.9 ± 0.6	23.8 ± 6.1	26.6 ± 4.7	19.0 ± 6.8	23.9 ± 4.7	13.2 ± 0.6
n	6	6	5	3	5	6	5	5	3	6
P	–	0.70	0.25	0.55	0.0043	–	0.54	0.66	0.71	0.13
**CQ+VP IC_{90}**	80.2 ± 7.2	62.3 ± 0.9	79.5 ± 8.7	66.6 ± 11.6	24.4 ± 2.4	78.8 ± 13.2	72.1 ± 12.8	71.7 ± 13.8	59.3 ± 8.11	26.4 ± 1.1
n	6	5	5	3	5	6	5	5	3	6
P	–	0.33	0.93	0.55	0.0043	–	0.54	0.43	0.71	0.0022
**% Reversibility CQ**	0.79 ± 0.02	0.79 ± 0.04	0.84 ± 0.04	0.70 ± 0.07	-0.08± 0.07	0.84 ± 0.03	0.78 ± 0.04	0.86 ± 0.04	0.74 ± 0.03	-0.03 ± 0.07
n	6	6	5	3	5	6	5	5	3	6
P	–	0.97	0.35	0.33	0.0043	–	0.27	0.71	0.19	0.0022
**md-CQ IC_{50}**	845 ± 44	703 ± 80	744 ± 69	537 ± 66	21.2 ± 0.8	720 ± 29	608 ± 56	602 ± 47	426 ± 53	21.0 ± 2.4
n	7	7	5	4	7	7	6	5	4	6
P	–	0.05	0.07	0.0061	0.0006	–	0.14	0.07	0.0061	0.0012
**md-CQ IC_{90}**	1527 ± 68	1309 ±83	1428 ± 58	828 ± 74	48.3 ± 1.7	1334 ± 134	1144 ± 137	1186 ± 109	742 ± 103	48.1 ± 3.3
n	7	7	5	4	7	7	6	5	4	6
P	–	0.13	0.53	0.0061	0.0006	–	0.45	0.43	0.01	0.0012
**md-ADQ IC_{50}**	90.7 ± 6.2	45.3 ± 2.9	71.5 ± 7.7	44.4 ± 6.8	23.7 ± 1.7	79.1 ± 3.8	46.5 ± 2.2	69.5 ± 2.2	43.5 ± 5.8	27.1 ± 1.9
n	7	7	5	4	7	7	6	5	4	6
P	–	0.0006	0.11	0.0061	0.0006	–	0.0012	0.11	0.0061	0.0012
**md-ADQ IC_{90}**	163 ± 8.7	88.9 ± 4.6	117 ± 13.8	79.7 ± 8.5	37.7 ± 2.7	131 ± 11.3	80.2 ± 6.6	122 ± 9.9	64.1 ± 12.3	36.7 ± 3.2
n	7	6	5	3	6	7	6	5	3	6
P	–	0.0012	0.03	0.02	0.0012	–	0.01	0.43	0.03	0.0012
**LMF IC_{50}**	1.1 ± 0.1	1.2 ± 0.2	1.0 ± 0.04	1.6 ± 0.5	2.0 ± 0.2	1.0 ± 0.2	1.2 ± 0.2	1.1 ± 0.2	1.1 ± 0.04	1.9 ± 0.4
n	4	7	4	3	6	4	4	4	3	4
P	–	0.34	0.86	0.40	0.0095	–	0.49	0.89	0.86	0.20
**LMF IC_{90}**	11.2 ± 1.1	8.9 ± 1.1	13.2 ± 1.3	16.0 ± 5.6	12.2 ± 1.2	9.1 ± 1.4	8.7 ± 1.4	7.0 ± 0.6	12.0 ± 7.1	9.2 ± 0.7
n	4	7	4	3	6	4	4	4	3	4
P	–	0.23	0.20	0.63	0.61	–	0.89	0.34	>0.99	0.89

Supplementary Table S5 (page 2 of 2). Antimalarial IC_{50} and IC_{90} values of *pfcrt*-modified parasite lines.

**AS IC_{50}**	2.3 ± 0.2	2.2 ± 0.3	2.5 ± 0.2	1.8 ± 0.2	2.3 ± 0.2	2.3 ± 0.3	2.5 ± 0.3	2.2 ± 0.2	1.7 ± 0.3	3.0 ± 0.2
n	7	9	6	5	10	7	6	6	5	6
P	–	0.84	0.29	0.26	0.83	–	0.54	0.83	0.43	0.1
**AS IC_{90}**	4.1 ± 0.3	4.7 ± 0.6	5.5 ± 0.7	4.2 ± 0.8	4.9 ± 0.5	4.6 ± 0.6	4.9 ± 0.6	5.0 ± 0.6	3.7 ± 1.0	5.7 ± 0.9
n	7	7	6	3	8	7	6	6	3	7
P	–	0.38	0.05	>0.99	0.19	–	0.63	0.63	0.67	0.26
**PPQ IC_{50}**	13.8 ± 0.9	12.4 ± 0.9	13.5 ± 1.0	14.7 ± 1.3	10.9 ± 1.3	11.8 ± 0.6	10.1 ± 0.6	9.9 ± 1.0	8.5 ± 0.4	15.7 ± 2.1
n	6	7	5	4	8	5	5	5	5	5
P	–	0.38	>0.99	0.71	0.05	–	0.15	0.22	0.0079	0.15
**PPQ IC_{90}**	29.7 ± 2.2	24.4 ± 2.9	29.0 ± 1.0	29.4 ± 5.0	22.1 ± 3.2	23.2 ± 3.4	19.8 ± 2.2	21.4 ± 3.1	17.1 ± 2.0	34.7 ± 5.4
n	6	7	5	4	8	5	5	5	5	5
P	–	0.23	0.30	0.44	0.04	–	0.69	0.55	0.22	0.55
**QN IC_{50}**	139 ± 22.5	134 ± 21.1	118 ± 15.6	128 ± 19.7	96.9 ± 8.2	98.1 ± 16.2	120 ± 15.0	103 ± 8.6	126 ± 43.0	83.7 ± 6.6
n	4	7	5	4	6	4	4	4	4	4
P	–	0.79	0.56	0.69	0.17	–	0.34	0.89	0.89	0.89
**QN IC_{90}**	355 ± 19	393 ± 58	363 ± 4	587 ± 169	318 ± 68	390 ± 25	436 ± 50	408 ± 59	399 ± 96	296 ± 42
n	4	7	5	4	6	4	4	4	4	4
P	–	0.93	0.91	0.89	0.48	–	0.69	0.89	0.69	0.11
**PND IC_{50}**	11.7 ± 2.1	10.8 ± 1.0	12.6 ± 1.5	10.2 ± 1.1	9.3 ± 1.6	11.8 ± 1.5	14.2 ± 1.3	13.6 ± 1.8	8.2 ± 3.0	14.0 ± 1.9
n	5	6	5	3	7	5	4	5	3	5
P	–	0.93	0.84	>0.99	0.43	–	0.41	0.55	0.39	0.42
**PND IC_{90}**	19.5 ± 4.1	17.0 ± 0.5	21.7 ± 3.5	17.8 ± 0.7	13.9 ± 2.4	20.1 ± 3.1	21.7 ± 4.0	21.0 ± 3.3	14.5 ± 5.6	19.3 ± 3.2
n	5	6	5	3	7	5	4	5	3	5
P	–	0.93	0.69	>0.99	0.43	–	0.56	0.69	0.86	0.93

IC_{50} and IC_{90} values (nM) indicate the mean ± SEM, as determined in 3 to 10 independent assays. CQ, chloroquine; VP, verapamil; md-CQ, monodesethyl-chloroquine; md-ADQ, monodesethyl-amodiaquine; LMF, lumefantrine; AS, artesunate; PPQ, piperaquine; QN, quinine; PND, pyronaridine; *n*, number of assays. CQ + VP assays were performed with 0.8 μM VP. Statistical significance was determined via two-tailed Mann Whitney *U* test. *P* values are reported for comparisons with the parasite line $Dd2^{Dd2}$ (left side) or $Dd2_{R539T}^{Dd2}$ (right side).

Color code:	ns	*p<0.05	**p<0.01	***p<0.001
