# Supplementary material for: Global Spread of Mutant PfCRT and Its Pleiotropic Impact on Plasmodium falciparum Multidrug Resistance and Fitness
Source: mBio. 2019 Apr 30;10(2):e02731-18. doi: 10.1128/mBio.02731-18 (PMC6495381; doi:10.1128/mBio.02731-18)
Supplement: TABLE S6 [file mBio.02731-18-st006.pdf]

Supplementary Table S6. *In vitro* growth selection coefficients of *pfcrt*-modified and reference parasite lines.

Line	**Dd2 parent**	**$Dd2^{Dd2}$**	**$Dd2^{Cam783}$**	**$Dd2^{FCB}$**	**$Dd2^{GB4}$**	**$Dd2^{3D7}$**
s	-0.12 ± 0.007	-0.16 ± 0.007	-0.04 ± 0.011	-0.20 ± 0.009	-0.08 ± 0.014	0.00 ± 0.012
P_1	<0.0001	<0.0001	0.03	<0.0001	<0.0001	–
Line	**$Dd2_{R539T}$ parent**	**$Dd2_{R539T}^{Dd2}$**	**$Dd2_{R539T}^{Cam783}$**	**$Dd2_{R539T}^{FCB}$**	**$Dd2_{R539T}^{GB4}$**	**$Dd2_{R539T}^{3D7}$**
s	-0.24 ± 0.007	-0.24 ± 0.006	-0.09 ± 0.010	-0.22 ± 0.006	-0.07 ± 0.01	-0.05 ± 0.01
P_1	<0.0001	<0.0001	0.01	<0.0001	0.54	–
P_2	<0.0001	<0.0001	0.002	0.57	0.96	<0.0001

Growth selection coefficients (s) of recombinant and parental parasite lines were determined as detailed in **Supplementary Materials and Methods**, and were normalized against the $Dd2^{3D7}$ line that expresses the wild-type (3D7) *pfcrt* allele. *s* <0 and *s* >0 indicate growth inferior or superior to the $Dd2^{3D7}$ line, respectively. Listed are mean *s* ± SEM values, which encompass three independent experiments performed in duplicate (*n* =6 total replicates per line). Statistical comparisons were done using a two-way ANOVA with Sidak's post-hoc test. *P* indicates the statistical significance for *pfcrt*-variant lines as compared to otherwise isogenic parasites encoding 3D7 *pfcrt* (either $Dd2^{3D7}$ or $Dd2_{R539T}^{3D7}$). P_2 indicates the statistical significance observed between *pfcrt*-matched lines in the Dd2 (wild-type K13) and $Dd2_{R539T}$ (mutant K13) genetic backgrounds.

Color code	ns	*p<0.05	**p<0.01	***p<0.001
